# Study on the Antibreast Cancer Mechanism and Bioactive Components of *Si-Wu-Tang* by Cell Type-Specific Molecular Network

**DOI:** 10.1155/2020/2345970

**Published:** 2020-03-12

**Authors:** Baixia Zhang, Rao Zheng, Yun Wang

**Affiliations:** ^1^College of Chinese Medicine, Hebei University, Hebei, Baoding 071002, China; ^2^College of Mechanical and Electronical Engineering, Beijing University of Chemical Technology, Beijing 100029, China; ^3^School of Chinese Materia Pharmacy, Beijing University of Chinese Medicine, Beijing 102488, China

## Abstract

Si-Wu-Tang (*SWT*), a traditional Chinese herbal formula, has shown an effect on antibreast cancer. However, the mechanisms and bioactive components of *SWT* are still unclear. Fortunately, cell type-specific molecular network has provided an effective method. This study integrated the data of formula components, all types of biomolecules in the human body, and nonexpressed protein in breast cancer cells and constructed the breast cancer cell network and the biological network that *SWT* acted on the breast cancer-related targets by Entity Grammar System (EGS). Biological network showed 59 bioactive components acting on 15 breast cancer-related targets. The antibreast cancer mechanisms were summarized by enrichment analysis: regulation of cell death, response to hormone stimulation, response to organic substance, regulation of phosphorylation of amino acids, regulation of cell proliferation, regulation of signal transmission, and affection of gland development. In addition, we discovered that verbascoside played the role of antibreast cancer by inhibiting cell proliferation, but there was not a report on this effect. The results of CCK8 and western blot were consistent with the antibreast cancer effect of verbascoside based on biological network. Biological network modeling by EGS and network analysis provide an effective way for uncovering the mechanism and identifying the bioactive components of *SWT*.

## 1. Introduction

Traditional Chinese medicine (TCM) formula, a herb combination, has shown a better effect on treating complex diseases [1, 2], such as diabetes, hypertension, and cancer. In the TCM formula, each herb contains numerous components that offer multitarget, multicomponent synergy, and multidimensional pharmacological actions. Besides, some diseases are related to more than one target [3]. Taken together, there is a considerable challenge for researchers to study the mechanisms and bioactive components of the formula using conventional pharmacological methods.

Fortunately, with the improvement of molecular biology and pharmaceutical chemistry, more and more public databases related to biomolecule and TCM have been constructed. Meanwhile, the large-scale molecular detection in various cells and tissues provides abundant omics data for network construction with the improvement of high throughput detection technologies. And above all the emergence of cell type-specific molecular network provided novel idea and orientation for the mechanism and bioactive components study of TCM formula [4, 5].

*Si-Wu-Tang* (*SWT*), a TCM formula, which consists of four herbs including Rehmanniae Radix Praeparata (*Rehmannia glutinosa* Libosch.), Angelicae Sinensis Radix (*Angelica sinensis* (Oliv.) Diels), Paeoniae Radix Alba (*Paeonia lactiflora* Pall.), and Chuanxiong Rhizoma (*Ligusticum chuanxiong* Hort.), is used for treatment of breast cancer especially the terminal breast cancer [6–8]. However, the antibreast cancer mechanism and bioactive components of *SWT* have not yet been systematically analyzed. This study integrated the data of TCM formula components, targets of components, metabolism data, gene-transcription factors, biological reactions, and signaling pathways and constructed the breast cancer cell network and the biological network that *SWT* acted on the breast cancer-related targets by Entity Grammar System (EGS). The biological network could clearly show the interaction between bioactive components of *SWT* and biomolecules of breast cancer cells. Through network and enrichment analysis, we could identify the bioactive components and summarize the antibreast cancer mechanism of *SWT*. Through single pathway analysis, we found a novel antibreast cancer ingredient-verbascoside. CCK8 and western blot were applied to test the antibreast cancer effect and the specific biological pathway of bioactive components.

## 2. Methods and Materials

### 2.1. Data Sources

The components of four herbs in *SWT* were collected from TCM Database@Taiwan [9] and TCMSP [10]; then the components were supplemented and perfected by the literature in CNKI and PubMed (1979∼2019). The name, structure, and SMILES string of components should be recorded. Repetitive components were deleted by name. For components which possess synonym, repetitive components were deleted by structural similarity. The collected components in each herb of *SWT* can be seen in Supplemental [Supplementary-material supplementary-material-1].

The proteins which could interact with components were derived from STITCH 5.0 [11] and DrugBank version 5.1.4 [12]. In STITCH, the proteins which could interact with components were discovered by name or SMILES string. The required confidence was higher than 0.400 and the active prediction methods included gene fusion, cooccurrence, coexpression, experiments, databases, neighborhood, predictions, and text mining. Because some components could not find their targets in STITCH, we found them by molecular similarity in DrugBank. The similarity threshold was 0.7 and the other parameters values were “default.” The proteins which could interact with the components were recorded in Supplemental [Supplementary-material supplementary-material-1].

The data of biological reactions and signaling pathways were derived from the Reactome [13]. Metabolism data of small molecules was from KEGG [14] and HumanCyc [15]. The data of gene-transcription factors was derived from TRRD [16] and TRED [17]. The data of specific expression proteins was derived from the Gene Expression Omnibus (GSE23610) [18]. Breast cancer-related targets (Supplemental [Supplementary-material supplementary-material-1]) were collected from TTD [19] and DrugBank version 4.3.

### 2.2. The Construction of Breast Cancer Cell Network and the Biological Network That SWT Acted on the Breast Cancer-Related Targets by EGS

How to integrate the fragmentary information and further much useful knowledge is a crucial work. So a method which could model the interactions of molecules in biological systems and simulate the evolution of systems is required. In this study, we used EGS to construct the breast cancer cell network and the biological network that *SWT* acted on the breast cancer-related targets.

EGS is a quadri-tuple, *G* = (*V*, *F*, *P*, *S*), whereas *V* is the character set representing a basic element, *F* is a finite set of relations for *V*, *V* and *F* were viewed as entity, *P* is a set of rules to deduce relationships between entities, and S is the starting entity [20]. According to different objective, we can write different reasoning engine. Providing a starting condition (starting entity, such as components in herb or TCM formula), EGS can obtain the result of the relationship among entities automatically. With these relationships among entities, we can construct formula-biomolecule interaction networks in a specific cell. Here, we used EGS as a framework to find the relationships between components and other entities. The results, after reasoning and rearranging, were visualized with Cytoscape. Thus, we could construct a cell type-specific molecular network. The application of EGS is more flexible compared to traditional network modeling methods. According to different objectives, we can define different *V*, *F*, *P*, and *S*.

The *V*, *F*, *P*, and *S* of the breast cancer cell network and the biological network that *SWT* acted on the breast cancer-related targets were described by the following:

(1) *V*=*V*_1_ ∪ *V*_2_  ∪  *V*_3_  ∪  *V*_4_  ∪  *V*_5_  ∪  *V*_6_  ∪  *V*_7_  ∪  *V*_8_
   
*V*_1_ is the set of components of the corresponding herb, *V*_2_ is the set of proteins which can interact with components, *V*_3_ is the set of breast cancer-related proteins, *V*_4_ is the set of rest proteins in the whole biological network of the human body, and *V*_5_ is the set of rest complexes or small molecule metabolites in the whole biological network of the human body. *V*_6_ is the set of none-express proteins in breast cancer cells, *V*_7_ is the set of the number of reactions or metabolisms, and *V*_8_ is the set of chemical constituents.
(2) *F* = {reaction (*A*, *B*, *C*, *D*, *E*), metainput (*A*, *B*, *C*), metaoutput (*A*, *B*, *C*), metaenzyme (*A*, *B*, *C*, *D*), tf (*A*, *B*, *C*, *D*, *E*, *F*), noneexpress (*A*, *B*, *C*), biochemreac (*A*), metabolism (*A*), notf (*A*), link (*A*, *B*, *C*), draw (*A*, *B*, *X*, *Y*), forward (*A*, *B*, *X*, *Y*), backward (*A*, *B*, *X*, *Y*), start (*A*, *B*), dis (*A*, *B*), pict (*A*, *B*, *X*, *Y*), length (*Y*)}.
In reaction (*A*, *B*, *C*, *D*, *E*), *A* ∈ *V*_4_  ∪  *V*_5_, *D* ∈ *V*_7_, *E* ∈ {pos, neg, known, enzyme}. It defines that *A* acts as the reactant or product of the biochemical reaction *D* with the action of *E*. In metainput (*A*, *B*, *C*), metaoutput (*A*, *B*, *C*), *A* ∈ *V*_8_, *B* ∈ *V*_7_, *C* represents the reactant or product of the metabolic reaction of *B*. In metaenzyme (*A*, *B*, *C*, *D*), *A* represents the enzyme of metabolic reaction *D*. The tf (*A*, *B*, *C*, *D*, *E*, *F*) defines that A acts as the transcript factor of *C* with the action of *F*. The noneexpress (*A*, *B*, *C*) defines that protein *A* is not expressed in cell *B* and labels *C* as “none.” In biochemreac (*A*), no tf (*A*), and metabolism (*A*), *A* labels the number of biochemical reactions, metabolism reactions, or transcriptions. In link (*A*, *B*, *C*), *A*, *B* ∈ *V*_4_  ∪  *V*_5_  ∪  *V*_8_, *C *∈* *{pos, neg, known, enzyme}. The link (*A*, *B*, *C*) defines the interaction of all types of molecules which exist in the whole biological network of the human body network. Draw (*A*, *B*, *C*, *Y*), forward (*A*, *B*, *C*, *Y*) and backward (*A*, *B*, *C*, *Y*), *A*, *B* ∈ *V*_4_  ∪  *V*_5_  ∪  *V*_8_, *C *∈* *{pos, neg, known, enzyme}, *Y* ∈ *Z*^*∗*^. That is, *A* acts on *B* with an effect described in *C* through *Y* reactions and the distance number is *Y*. If a process takes place in one reaction, *Y* equals 1. The start (*A*, *B*), *A* ∈ *V*_1_, *B* ∈ *V*_2_. In other words, *A* represents the components of the corresponding herbs; *B* represents the targets of compounds. In dis (*A*, *B*), *A* represents the disease-breast cancer, while *B* represents the breast cancer-related proteins. In pict (*A*, *B*, *C*, *Y*), *A* ∈ *V*_2_, *B* ∈ *V*_3_, *C* ∈ {pos, neg, known, enzyme}, *Y* ∈ *Z*^*∗*^. The pict (*A*, *B*, *C*, *Y*) represents that *A* (targets of components) affects *B* (breast cancer-related protein) with an effect described in *C* by the reactions of *Y*. One thing to note here is that the same letters may represent different meaning in various entities; different letters represent the same meaning because they are located in the same entity.
(3) *P*=*P*_1_  ∪  *P*_2_  ∪  *P*_3_  ∪  *P*_4_  ∪  *P*_5_  ∪  *P*_6_  ∪  *P*_7_  ∪  *P*_8_  ∪  *P*_9_  ∪  *P*_10_  ∪  *P*_11_  ∪  *P*_12_  ∪  *P*_13_  ∪  *P*_14_  ∪  *P*_15_  ∪  *P*_16_
*P*_1_ = {reaction (*A*, *D*, *E*, *F*, *G*), noneexpress (*A*, *B*, *C*) ⇒ biochemreact (*F*)},
*P*_2_ = {not biochemreac (*J*), reaction (*G*, *H*, *I*, *J*, *K*) ⇒ link (*G*, *H*, *K*)},
*P*_3_ = {metaenzyme (*A*, *D*, *E*, *F*), noneexpress (*A*, *B*, *C*) ⇒ metabolism (*F*)},
*P*_4_ = {metaenzyme (*A*, *D*, *E*, *G*), not metabolism (*G*) ⇒ link (*A*, *D*, *G*)},
*P*_5_ = {metainput (*G*, *H*, *I*), not metabolism (*I*) ⇒ link (*G*, *H*, *I*)},
*P*_6_ = {metaoutput (*G*, *H*, *I*), not metabolism (*I*) ⇒ link (*G*, *H*, *I*)},
*P*_7_ = {tf (*A*, *D*, *E*, *F*, *G*, *H*), noneexpress (*A*, *B*, *C*) ⇒ notf (*H*)},
*P*_8_ = {tf (*I*, *J*, *K*, *L*, *M*, *N*), not notf (*N*) ⇒ link (*I*, *K*, *M*)},
*P*_9_ = {link (*A*, *B*, *X*), start (*A*) ⇒ draw (*A*, *B*, *X*, *M*)},
*P*_10_ = {draw (*A*, *B*, *X*, *N*), link (*B*, *C*, *D*), *M* = *N* + 1, *N* < 15 ⇒ draw (*A*, *C*, *D*, *M*)},
*P*_11_ = {draw (*C*, *E*, *F*, *X*), dis (*A*, *D*), *E* = *D*⇒pict (*C*, *D*, *F*, *X*)},
*P*_12_ = {pict (*C*, *B*, *X*, *Y*) ⇒ length (*Y*)},
*P*_13_ = {link (*C*, *B*, *D*), pict (_, *B*, _, _) ⇒ backward (*C*, *B*, *D*, 1)},
*P*_14_ = {link (*D*, *C*, *E*), backward (*C*, *B*, *D*, *N*), *M* = *N* + 1, *N* < *Y*, length (*Y*) ⇒ backward (*D*, *C*, *E*, *F*)},
*P*_15_ = {pict (*A*, _, _, _), backward (*A*, *C*, *X*, *Y*) ⇒ forward (*A*, *C*, *X*, 1)},
*P*_16_ = {forward (*A*, *C*, *X*, *N*), backward (*C*, *D*, *E*, *F*), *M* = *N* + 1, *N*=*Y* *−* *F*, length (*Y*) ⇒ forward (*C*, *D*, *E*, *M*)}.
   
*P*_1_  ∪  *P*_2_  ∪  *P*_3_  ∪  *P*_4_  ∪  *P*_5_  ∪  *P*_6_  ∪  *P*_7_  ∪  *P*_8_ is a set of rules which were utilized to construct the breast cancer cell network. *P*_9_  ∪  *P*_10_  ∪  *P*_11_ was utilized to compute all the numbers of disease-related proteins that the components could affect and the distance between them. The distances were limited within 15 steps. *P*_9_ labels the starting point and *P*_11_ labels the ending point. *P*_12_  ∪  *P*_13_  ∪  *P*_14_  ∪  *P*_15_  ∪  *P*_16_ were used to describe the detailed pathway from components target to disease-related proteins, with the specific steps of *Y.* Forward (*C*, *D*, *E*, *M*) was the final result used to construct the network. After connecting each of the forward (*C*, *D*, *E*, *M*), we could get the detailed pathway from components to disease-related proteins.
(4) *S*=*S*_1_  ∪  *S*_2_
   
*S*_1_ is the set of entities in the biological network of the whole human body for deduction, such as reaction (*A*, _, _*D*, *E*), noneexpress (*A*, *B*, *C*), biochemreac (*F*), metaenzyme (*A*, *B*, *C*, *D*), metabolism (*A*), metainput (*A*, *B*, *C*), metaoutput (*A*, *B*, *C*), tf (*A*, _, _*D*, *E*, *F*), and link (*A*, *B*, *X*). *S*_2_ is the set of starting and ending point proteins and was described by start (*A*, *B*) and dis (*A*, *B*). In this study, the starting points were defined as none-express proteins in breast cancer cells and components in *SWT*, respectively. The ending point was defined as breast cancer-related proteins when constructing the biological network that *SWT* acted on the breast cancer-related targets.

### 2.3. The Bioactive Components of SWT

Starting with a disease target, this study could identify the bioactive components of *SWT* reverse biological pathway. In other words, the letter *C* in forward (*C*, *D*, *E*, *M*) represents bioactive components.

### 2.4. Enrichment Analysis

Gene-GO term enrichment analysis was utilized to highlight the most relevant biopathways associated with a given gene list. It was carried by DAVID 6.7 Functional Annotation Clustering [21]. Values of *p* < 0.05 and *FDR *<* *0.05 were considered as significantly enriched biopathways.

### 2.5. CCK8 Was Used to Detect the Influence of Verbascoside on the Proliferation Activity of MCF-7

Verbascoside and paclitaxel were purchased from the National Institutes for Food and Drug Control (Beijing, China). High DMEM basal medium was purchased from HyClone® (Beijing, China). FBS was purchased from GIBCO®/Invitrogen Life Technologies (Beijing, China). Double-antibody was purchased from ProSpec® (Beijing, China).

The human breast cancer cell line MCF-7 was obtained from Jiamay Biotech Co., Ltd. (Beijing, China). The MCF-7 cells of logarithmic growth were inoculated in 96-well plates (5000/well). Holes without cells were used as blank controls. The plate was preincubated at 37°C in a humidified atmosphere comprised of 95% air and 5% CO_2_. Verbascoside was diluted in DMSO and then added to each well at a final concentration of 0, 5, 10, 20, 40, 80, and 160 *μ*M, which were incubated for 24 h, 48 h, and 72 h. Paclitaxel (3.83 *μ*g/mL) was used as positive control group. The above groups were all set 5 repeats. CCK-8 solution (10 *μ*L) was added to each well of the plates. The plates was incubated for 1∼4 h. The absorbance was measured at 450 nm using a microplate reader (Thermo).

Cell survival rate = *A*_1_ (medication group)/*A*_2_ (blank control group) ^*∗*^100%; the results were shown as the mean ± standard error of the mean (SEM). The differences between the two groups of independent samples were analyzed by Student's *t*-test. *p* < 0.05 was considered statistically significant. The IC50 in different timings were calculated by GraphPad Prism 6.

### 2.6. Western Blot Was Used to Detect the Effluences of Verbascoside on the Expression of Cdk6, AP-1, and MYC in MCF-7

The MCF-7 cells of logarithmic growth were inoculated in 96-well plates (5000/well). Holes without cells were used as blank controls. The plate was preincubated at 37°C in a humidified atmosphere comprised of 95% air and 5% CO_2_. Verbascoside was diluted in DMSO and then added to each well at a final concentration of 0, 1, 2, 4, 8, 16, and 32 *μ*M, which were incubated for 48 h. Paclitaxel (3.83 *μ*g/mL) was used as positive control group. The above groups were all set 5 repeats. Cell lysis buffer was prepared by PMSF, cocktail, Triton-100, NaVO3, and base lysis buffer. Protein lysis buffer (150 *μ*L) was added to the cells of black control group, paclitaxel group, and verbascoside group, vortex 8∼10 times, pyrolysis 35∼40 min on ice; The lysates were then centrifuged at 12000 rpm for 15 min at 4°C; the concentration of the total proteins was determined using BCA kit. The proteins were separated by SDS-polyacrylamide gel electrophoresis (SDS-PAGE) and then were transferred into a PVDF membrane. The membranes were blocked with 5% skimmed milk in TBST for 2 h at room temperature and incubated with specific primary antibodies overnight at 4°C. The membranes were washed three times and incubated with appropriate secondary antibodies within 1 h at room temperature. Protein bands were detected using the chemiluminescence detection system (Applygen, China). All of the western blots were performed at least three times.

The results were shown as the mean ± standard error of the mean (SEM). The differences between the two groups of independent samples were analyzed by Student's *t*-test. *p* < 0.05 was considered statistically significant.

## 3. Results and Discussions

### 3.1. The Breast Cancer Cell Network

**Figure 1:**
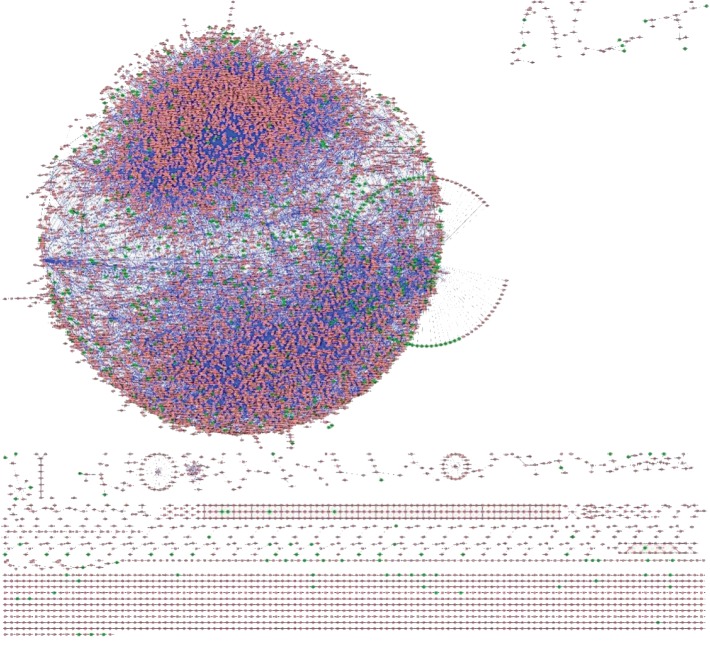
The breast cancer cell network. Green nodes represented differentially expressed genes. Red nodes represented other types of biomolecules involved in the breast cancer cell.

With none-express proteins in the breast cancer cell as the starting point, all the biological reactions, that is, link (*I*, *K*, *M*), in the breast cancer cell, were visualized with Cytoscape. We could obtain the breast cancer cell network (Figure 1). This network can be used as the background network to qualitatively deduce the antibreast cancer mechanism of *SWT*.

### 3.2. The Biological Network That SWT Acted on Breast Cancer-Related Targets by EGS

**Figure 2:**
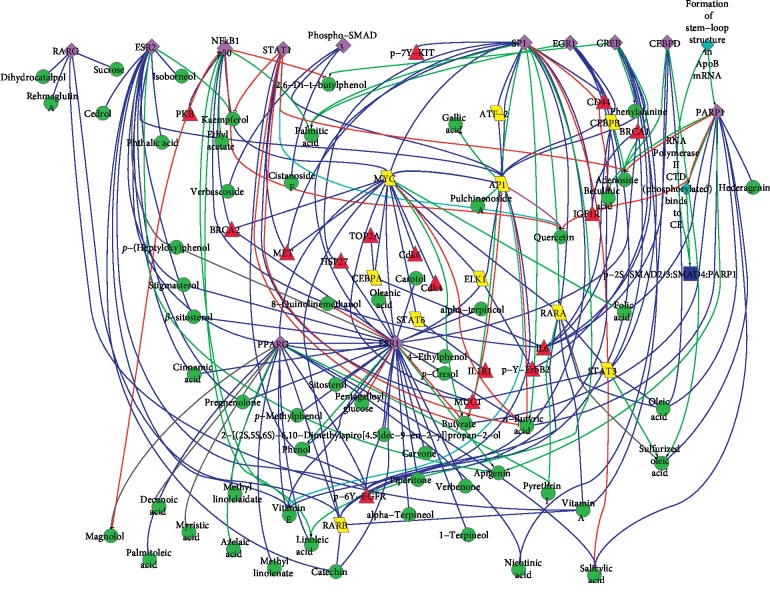
The biological networks of *SWT* that acted on breast cancer-related targets. This network displayed all the pathways that bioactive components acted on the breast cancer-related targets. Different shapes gave the nodes various biological roles. Red node represented breast cancer-related targets, green node represented bioactive components, purple node represented the proteins which can interact with bioactive components, yellow node represented genes and transcript factors, blue node represented complex, and bright blue node represented biological reaction. The green edge represented positive regulation, red edge represented negative regulation, the gray edge represented binding, and the blue edge represented the direction which is uncertain.

With all the components as the starting point and breast cancer-related targets as the ending point, the biological networks of *SWT* that acted on the breast cancer-related targets were constructed by EGS and visualized by Cytoscape (Figure 2).

Figure 2 could overall exhibit the bioactive components and the antibreast cancer mechanism on a molecular level. One component could act on multiple targets and multiple components could act on only one target. *SWT* played a regulatory role through regulating multiple components, multiple targets, and multiple pathways. These illustrated that the targets and pathways of *SWT* were extensive. That is to say, its targets and pathways were not specific but comprehensive. Besides, some components could act on one target, but the active directions were opposite. For example, the direction of quercetin acted on NFκB1 and p50 was positive, while palmitoleic acid was negative. *SWT* may keep the cell state in balance through this opposite regulation.

### 3.3. The Bioactive Components of SWT

The biological networks clearly showed the 15 breast cancer-related targets affected by 59 bioactive components. These 59 components (Table 1) were considered as bioactive components of *SWT*. These indicated that multiple components of *SWT* play an antibreast cancer role through multiple biological pathways and targets.

According to the results computed by EGS, the biopathway of each bioactive component could be extracted and described. For example, kaempferol could inhibit the proliferation of MCF-7 by increasing the expression of ESR2, improving the activity of ESR2 and PPARG, and finally inhibiting EGFR activity.

### 3.4. Enriched Pathways of Breast Cancer-Related Targets Affected by SWT

Single biopathway affected by bioactive components could only illustrate the antibreast cancer mechanism of *SWT* in one aspect. Enrichment analysis of breast cancer-related targets affected by *SWT* could uncover the mechanism comprehensively. These targets were highlighted in 17 biopathways (Table 2).

We find that 17 biopathways could be divided into 7 groups. In other words, the antibreast cancer mechanism of *SWT* could be summarized as follows: response to organic substances, regulation of amino acid phosphorylation, regulation of cell death, response to hormone stimulation, regulation of cell proliferation, regulation of signal transmission, and affection of gland development. Apparently, they are all closely associated with the occurrence and development of breast cancer [22–28].

Some reversed regulations existed in the enrichment analysis of *SWT*, such as negative regulation of programmed cell death and positive regulation of cell proliferation. These pathways appeared to be opposite to antibreast cancer, but this phenomenon was also reasonable. They may be derived from the opposite active direction of some components. Some components acted on one target with the same or opposite active direction, such as quercetin and palmitoleic acid. These various directions may represent the synergism or antagonism between components. Just because of the synergism and antagonism of these bioactive components, *SWT* could maintain the homeostasis of the cell. This may be the reason why the drug combination had the potential to enhance efficacy and reduce toxicity [29, 30]. All the results fully demonstrated the curative properties of the TCM formula.

### 3.5. Biopathway Affected by Verbascoside

**Figure 3:**
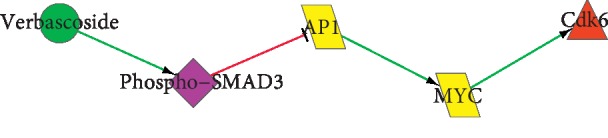
The biopathway of verbascoside acting on Cdk6. The meaning of each node and edge is the same as shown in Figure 2.

In order to clearly show and analyze the antibreast cancer mechanism of *SWT*, we extracted all the biopathways. The following was the biopathway that verbascoside acted on Cdk6 (Figure 3).

Verbascoside is the index component of Rehmanniae Radix Praeparata. It has some effects such as being neuroprotective [31], antipneumonia [32], antifatigue [33], being cardiotonic [34], etc. Research showed that verbascoside could have competitive binding estrogen receptors in Hela with estradiol, antagonizing the upregulation of ERE luciferase activity by estradiol. The effect of verbascoside is similar to known phytoestrogens such as resveratrol [35], indicating that verbascoside has plant estrogenic activity. In addition, verbascoside is associated with the proliferation and differentiation of colorectal cancer and gastric cancer cells [36, 37], but there are no reports on its effect on antibreast cancer and mechanism. We further verified the antibreast cancer effect of verbascoside in Sections 3.6 and 3.7.

From Figure 3, we could see that verbascoside enhanced the phosphorylation of SMAD3, promoted the expression of AP-1, and inhibited the expression of MYC and Cdk6. SMAD3 could control gene activity and inhibit cell proliferation; Ap-1 could control many cell processes, including differentiation, proliferation, and apoptosis. AP-1 inhibited the expression of cellular adhesion proteins by the phosphorylation of SMAD3 and inhibited nuclear transcription factor AP-1 in the biological pathway SMAD3/AP-1. Enhancing the activity of AP-1 could reduce the expression of MYC, and MYC was an oncogene that was closely related to the cell cycle. MYC was involved in the malignant transformation of human and animal cells and overexpression in many malignant tumors, which could lead to apoptosis of cells such as HL60 [38, 39]. The degradation of MYC could reduce the expression of Cdk6 [40]. Cdk6 was a cyclin-dependent kinase that controlled cell cycle; Cdk6 could make positive regulation of cell proliferation during the G1/S phase transition. In conclusion, verbascoside may play the role of antibreast cancer by inhibiting cell proliferation and promoting apoptosis. This study also tested the effect of antibreast cancer on CCK8 and western blot.

### 3.6. The Influence of Verbascoside on the Proliferation Activity of MCF-7

**Figure 4:**
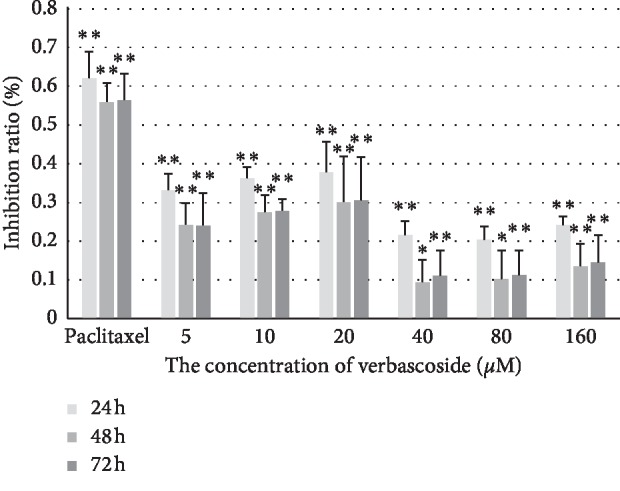
The influence of paclitaxel and verbascoside on the proliferation of MCF-7. Cells were treated with the indicated concentrations of paclitaxel (3.83 *μ*g/mL) and verbascoside for 24, 48, and 72 h. The viabilities of cells were evaluated by CCK8 assay. ^*∗*^*p* < 0.05 and ^*∗∗*^*p* < 0.01 compared to the control.

Figure 4 showed that the positive drug has the highest inhibition rate, and the inhibition rate was 56%∼62%. The inhibition rate of verbascoside was lower than paclitaxel. The highest inhibition rate of verbascoside was 30%∼38% when its concentration was 20 *μ*M. When the concentrations of verbascoside were 5∼20 *μ*M and 40∼160 *μ*M, its inhibition rate increased; when the concentration is 20∼40 *μ*M, its inhibition rate decreased. All these explained that verbascoside could inhibit the proliferation of MCF-7 at lower concentrations.

The IC50 of verbascoside in 24 h, 48 h, and 72 h were, respectively, 8.85, 4.405, and 4.565 *μ*M. Verbascoside had the best inhibition of proliferation at 24 h, and the inhibition rate decreases at 48 h and 72 h. To sum up, this study chose the concentrations of 2 *μ*M, 4 *μ*M, 8 *μ*M, 16 *μ*M, 32 *μ*M, and 24 h to carry out the western blot.

### 3.7. The Effluences of Verbascoside on the Expression of Cdk6, AP-1, and MYC in MCF-7

**Figure 5:**
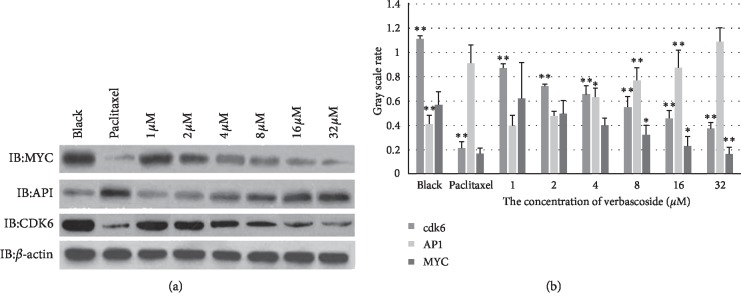
The influence of paclitaxel and verbascoside on the expression of Cdk6, AP-1, and MYC. Cells were incubated with the indicated concentrations of paclitaxel (3.83 *μ*g/mL) and verbascoside (0, 1, 2, 4, 8, 16, and 32 *μ*M) for 48 h; the cells were collected for western blotting as described in Materials and Methods. The results from 3 independent measurements are shown as the means ± SEM. ^*∗*^*p* < 0.05 and ^*∗∗*^*p* < 0.01 compared to the control.

From Figure 5, we could see that the expression of AP-1 increased, and the expression of Cdk6 and MYC decreased. That is to say, verbascoside facilitated the expression of AP-1, inhibited the expression of MYC and Cdk6, and achieved the optimum state at 32 *μ*M. Verbascoside and paclitaxel had the same influence tendency on the expression of the three proteins. The results of Figure 5 were consistent with the analysis in Figure 3.

In conclusion, verbascoside's antibreast cancer action mechanism enhanced the phosphorylation of SMAD3, promoted expression of AP-1, inhibited expression of MYC and Cdk6, and played the role of antibreast cancer by inhibiting the proliferation of MCF-7. The experimental results were consistent with the antibreast cancer effect of verbascoside based on biological network. All these verified the reliability of the bioactive components identification method based on cell type-specific molecular network.

## 4. Conclusion

The biological network constructed by EGS could exhibit the bioactive components of *SWT* and its antibreast cancer mechanism. 59 bioactive components of *SWT* act on 15 breast cancer-related targets. Each component of *SWT* plays a synergism or antagonism role through acting on similar biopathways and thus maintains the homeostasis of the cell. The antibreast cancer mechanisms of *SWT* were summarized by enrichment analysis as follows: regulation of cell death, response to hormone stimulation, response to organic substance, regulation of phosphorylation of amino acids, regulation of cell proliferation, regulation of signal transmission, and affection of gland development.

Single pathway could elucidate the antibreast cancer mechanism of each component. In this study, we identified and validated a novel antibreast cancer ingredient-verbascoside. The results of CCK8 and western blot verified the reliability of the bioactive components identification method based on cell type-specific molecular network. This study will provide the basis for the quality control of *SWT* and the design of combination drugs based on *SWT*.

Comparing to protein-protein interaction network (PPI), cell type-specific molecular network constructed by EGS is belonging to a multimolecular type network. EGS could integrate the information of gene regulation, metabolic network, cell signal, etc. Effectively, realizing batch processing is an efficient and flexible information fusion method. When combined with experimental data, especially all kinds of omics data, the space scale characteristics of complex systems are fully considered. At present, no studies have been reported on the mechanism of action of TCM using cell type-specific molecular networks. This study was based on the data included in the existing database or literature, and we did not take into account the quantity of components, so this method still has some limitations. With further study of the bioactivity of *SWT* components and breast cancer-related biomolecules, the data we used will be more complete and the results will be more precise and integrated.

## Figures and Tables

**Table 1:** The bioactive components of *SWT*.

Bioactive components	CAS ID	Bioactive components	CAS ID
2,6-Di-t-butylphenol	128-39-2	4-Ethylphenol	105-79-3
Betulinic acid	472-15-1	Azelaic acid	123-99-9
Cedrol	77-53-2	Decanoic acid	187997-16-6
Ethyl acetate	141-78-6	Magnolol	528-43-8
Gallic acid	149-91-7	Methyl linolenate	301-00-8
Hederagenin	465-99-6	Nicotinic acid	59-67-6
Kaempferol	520-18-3	Phthalic acid	88-99-3
Methyl linolelaidate	2566-97-4	*p*-Methylphenol	2186-24-5
Oleanic acid2hh	508-02-1	Quercetin	117-39-5
Pentagalloyl glucose	14937-32-7	Vitamin E	59-02-9
Pulchinenoside A	129724-84-1	Cistanoside F	97411-47-7
Pyrethrin I	121-21-1	Dihydrocatalpol	2415-24-9
Salicylic acid	7681-06-3	Palmitoleic acid	373-49-9
*β*-Sitosterol	83-46-5	Rehmaglutin A	103744-82-7
1-Terpineol	586-82-3	Verbascoside	84744-28-5
2-[(2S, 5S, 6S)-6, 10-Dimethylspiro[4.5]dec-9-en-2-yl]propan-2-ol	1460-73-7	Phenol	108-95-2
8-Quinolinemethanol	16032-35-2	Ferulic acid	537-98-4
Adenosine	30143-02-3	Folic acid	59-30-3
Alpha-terpineol	26531-51-1	Oleic acid	68609-92-7
Apigenin	520-36-5	*p*-Cresol	106-44-5
Carotol	465-28-1	Sitosterol	5779-62-4
Carvone	99-49-0	Verbenone	108-24-7
Catechin	139-85-5	Butyrate	123-72-8
Isoborneol	124-76-5	*n*-Butyric acid	107-92-6
*p*-(Heptyloxy)phenol	105-79-3	Sucrose	25702-74-3
Piperitone	89-81-6	Linoleic acid	60-33-3
Pregnenolone	145-13-1	Stigmasterol	83-48-7
Sulfurized oleic acid	60-33-3	Phenylalanine	673-31-4
Vitamin A	11103-57-4	Palmitic acid	1957-10-3

**Table 2:** Enriched pathways of breast cancer-related targets affected by *SWT.*

Term ID	Term name	*P* value	FDR
GO:0010033	Response to organic substance	8.44*E* − 11	1.31*E* − 07
GO:0043067	Regulation of programmed cell death	3.03*E* − 10	4.71*E* − 07
GO:0010941	Regulation of cell death	3.15*E* − 10	4.90*E* − 07
GO:0042981	Regulation of apoptosis	6.91*E* − 09	1.08*E* − 05
GO:0046777	Protein amino acid autophosphorylation	5.07*E* − 08	7.89*E* − 05
GO:0043069	Negative regulation of programmed cell death	1.35*E* − 07	2.10*E* − 04
GO:0060548	Negative regulation of cell death	1.38*E* − 07	2.15*E* − 04
GO:0009725	Response to hormone stimulus	1.57*E* − 07	2.45*E* − 04
GO:0009719	Response to endogenous stimulus	3.07*E* − 07	4.78*E* − 04
GO:0042127	Regulation of cell proliferation	1.92*E* − 06	0.002994
GO:0043066	Negative regulation of apoptosis	2.98*E* − 06	0.004645
GO:0007242	Intracellular signaling cascade	6.08*E* − 06	0.009468
GO:0007169	Transmembrane receptor protein tyrosine kinase signaling pathway	6.26*E* − 06	0.009747
GO:0008284	Positive regulation of cell proliferation	7.36*E* − 06	0.011458
GO:0006468	Protein amino acid phosphorylation	8.65*E* − 06	0.013465
GO:0048732	Gland development	2.04*E* − 05	0.031764
GO:0016310	Phosphorylation	2.84*E* − 05	0.044181

## Data Availability

The datasets used during the current study are available from the corresponding author on reasonable request.

## Supplementary Materials

Supplementary Materials Supplemental Information 1: 656 components of SWT. Supplemental Information 2: the proteins which could interact with the components of SWT. Supplemental Information 3: 60 breast cancer-related targets.
